# Effect of 35% hydrogen peroxide in promoting enamel whitening with orthodontic attachments bonded on its surface

**DOI:** 10.1590/2177-6709.30.3.e252547.oar

**Published:** 2025-10-20

**Authors:** Bruna Caroline Tomé BARRETO, Luiza Trindade VILELA, Guido Artemio MARAÑÓN-VÁSQUEZ, Matheus Melo PITHON, Margareth Maria Gomes de SOUZA

**Affiliations:** 1 Federal University of Rio de Janeiro, School of Dentistry, Department of Pediatric Dentistry and Orthodontics (Rio de Janeiro/RJ, Brazil).; 2 University of São Paulo, Ribeirão Preto School of Dentistry, Department of Pediatric Dentistry (Ribeirão Preto/SP, Brazil).; 3 Universidade Estadual do Sudoeste da Bahia, School of Dentistry, Department of Health (Jequié/BA, Brazil).

**Keywords:** Tooth whitening, Orthodontic appliances, Removable, Aesthetics, Clareamento dental, Aparelhos ortodônticos removíveis, Estética

## Abstract

**Introduction:**

The demand for aesthetics is high and the desire for white teeth during orthodontic treatment is a desire of patients.

**Objective:**

To evaluate the effect of 35% hydrogen peroxide in promoting enamel whitening with orthodontic attachments bonded to its surface.

**Methods:**

80 bovine incisors randomly divided into 4 groups (n=20): (GI) control group, (GII) group that received only tooth whitening, (GIII) group that received only bonding of attachments on the surface and (GIV) group that received bonding of attachments and whitening. Whitening was carried out following the manufacturer’s instructions while the attachments were bonded to the dental surface using a template after acid conditioning of the enamel and application of an adhesive system. Color evaluation was performed according to the LAB color scale of the Commission Internationale de l’Eclairage. Statistical analysis was performed using Jamovi program adopting a 5% significance level. Descriptive data analysis was performed, characterizing all study variables. General linear models adjusted by ordinary least squares regression were used to test the effect of whitening, enhancement use, and the interaction of both factors.

**Results:**

Both “whitening” and the use of “attachments” have an effect on all parameters of the CIELAB scale (keeping the other variable constant). The whitening effect was independent of the presence of attachments.

**Conclusion:**

35% hydrogen peroxide is capable of promoting enamel whitening with orthodontic attachments bonded to its surface.

**Clinical Meaning:**

Despite being *in vitro* research, the study provides patients and professionals with the possibility of teeth whitening during orthodontic treatment with aligners.

## RESUMO

**Introdução:**

A exigência por estética e o desejo por dentes brancos durante o tratamento ortodôntico é um desejo dos pacientes.

**Objetivo:**

Avaliar o efeito do peróxido de hidrogênio a 35% na promoção do clareamento do esmalte com *attachments* ortodônticos colados em sua superfície.

**Métodos:**

80 incisivos bovinos divididos aleatoriamente em 4 grupos (n=20): (GI) grupo controle, (GII) grupo que recebeu apenas clareamento dental, (GIII) grupo que recebeu apenas colagem de *attachments* na superfície e (GIV) grupo que recebeu colagem de *attachments* e clareamento. O clareamento foi realizado seguindo as instruções do fabricante e os *attachments* eram colados à superfície dentária por meio de um *template* após condicionamento ácido do esmalte e aplicação de sistema adesivo. A avaliação da cor foi realizada de acordo com a escala de cores LAB da Commission Internationale de l’Eclairage. A análise estatística foi realizada no programa Jamovi adotando nível de significância de 5%. Foi realizada análise descritiva dos dados, caracterizando todas as variáveis do estudo. Modelos lineares gerais ajustados por regressão de mínimos quadrados ordinários foram utilizados para testar o efeito do clareamento, do uso de *attachments* e da interação de ambos os fatores.

**Resultados:**

As variáveis “clareamento” e “uso de *attachments*” afetam todos os parâmetros da escala CIELAB (mantendo a outra variável constante). O efeito clareador foi independente da presença de *attachments*.

**Conclusão:**

o peróxido de hidrogênio a 35% é capaz de promover o clareamento do esmalte com *attachments* ortodônticos colados em sua superfície.

**Relevância Clínica:**

Apesar de ser uma pesquisa *in vitro*, o estudo proporciona aos pacientes e profissionais a possibilidade de clareamento dental durante o tratamento ortodôntico com alinhadores.

## INTRODUCTION

The search for dental aesthetics has been constant nowadays.^1^ Therefore, tooth whitening is the most popular aesthetic treatment in dentistry.^1^ The dental market offers products that have hydrogen peroxide as an agent, regardless of the technique used (office and/or home).^2^ The diffusion that occurs in whitening was first described by Haywood in 1990 and consists of the ability of peroxide to diffuse through the tooth structure, generate free radicals and degrade the organic molecules responsible for the intrinsic or extrinsic coloring of the tooth structure.^3^

In the background of Orthodontics, patients have reported a growing concern about whiter teeth.^4^ As whitening therapy during orthodontic treatment is an elective procedure, in order to consider the aesthetic complaint and patient satisfaction,^5^ some studies have shown a positive effect on tooth whitening in orthodontic patients.^5^ ^-^ ^7^ In fixed orthodontic treatment, tooth whitening is a possible and effective reality and studies show that even with orthodontic brackets in position, teeth are whitened by 35% hydrogen peroxide.^5^

With the introduction of removable clear aligners in the 1990s, this possibility of orthodontic treatment significantly increased patient interest compared to fixed appliance therapy,^8^ ^-^ ^10^ reinforcing the aesthetic appeal of patients.^11^ The use of transparent removable appliances as a therapy requires the aid of some appliances, such as attachments, to intensify the orthodontic movement.^12^ The attachments are composed of polymerized composite on the surface of the tooth with several functions aiming at the greater effectiveness of the orthodontic movement.^13^

Given the current aesthetic appeal and the immediacy of results for patients and considering that the whitening material acts by diffusion, the present study aims to evaluate, in vitro, the effect of 35% hydrogen peroxide in promoting enamel whitening with orthodontic appliances bonded to its surface, with the aim of enabling treatment with aligners at the same time as tooth whitening.

## METHODOLOGY

Ethical approval was granted by the Animal Use Ethics Committee of the Health Sciences Center of Federal University of Rio de Janeiro (Brazil).

Sample size was calculated based on pilot study, considering effect size f=0.36, a=0.05, power=0.8, numerator df=1 and number of groups=4. The estimated size resulted in a total of 63 teeth. Considering the possibility of using non-parametric statistics and the occurrence of losses during the study, 25% was added, totaling 80 teeth (20 x group). The sample consisted of enamel surfaces of bovine incisors, that were divided into four groups (Table 1).

**Table 1:** Study chronology.

	GI	GII	GIII	GIV
	without attachment and whitening	without attachment and with whitening	with attachment and without whitening	with attachment and whitening
T0	Color Evaluation	Color Evaluation	Color Evaluation	Color Evaluation
			Bonding	Bonding
T1		Whitening		Whitening
07 days in artificial saliva				
T2		Whitening		Whitening
07 days in artificial saliva				
T3			Debonding	Debonding
	Color Evaluation	Color Evaluation	Color Evaluation	Color Evaluation

**Figure 1:**
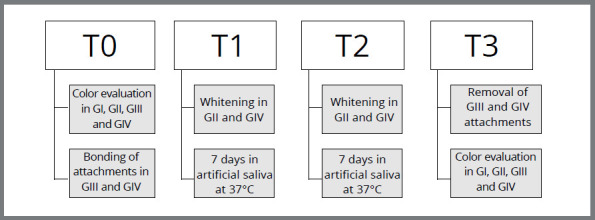
Study chronology.

As inclusion criteria, bovine dental enamel surfaces were used that were completely sound and without apparent defects, examined under halogen light. The chronology of the study is shown in Figure 1.

Specimens measuring 8 mm in diameter were made, individually fixed with adhesive wax on cuboidal acrylic plates, and the buccal surface of the crowns was sanded on the Politriz Metallographic machine (APL4, Arotec, Cotia, SP, Brazil) on a flat surface with running water. Following, they were stored in distilled water under refrigeration at 5°C until use.

Attachments (Vittra, A1, FGM, Joinville, Santa Catarina, Brazil) were manufactured using a prefabricated mold composed of addition silicone, in collaboration with a national company (Click Aligner, Rio Grande do Sul, Brazil). 

**Figure 2:**
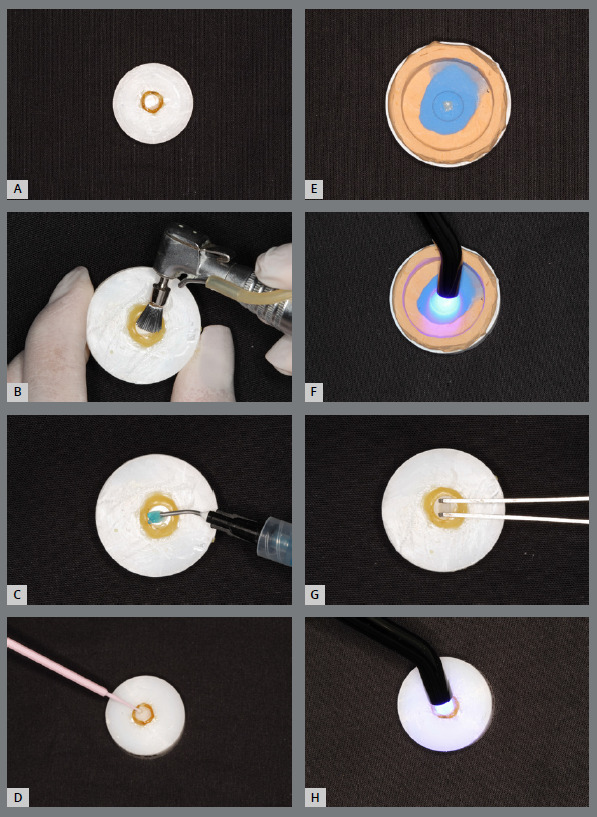
Enamel preparation sequence, attachment making and bonding. **A)** specimen; **B)** prophylaxis; **C)** application of 37% orthophosphoric acid; **D)** application of adhesive; **E)** composite resin inserted in the template; **F)**light curing; **G)** positioning of the attachment on the tooth; **H)** light curing.

Bovine dental enamel sections were subjected to prophylaxis, followed by conditioning with orthophosphoric acid (37%) (FGM, Joinville, Santa Catarina, Brazil), application of adhesive (Ambar Adhesive, FGM, Joinville, Santa Catarina, Brazil). The previously prepared template was positioned on the tooth surface followed by polymerization for 40 seconds with a power of 1100 mW/cm² (Kavo, Joinville, Santa Catarina, Brazil) (Fig. 2) To minimize the risk of gaps, the researcher carried out a visual inspection with a stereoscopic magnifying glass.

**Figure 3:**
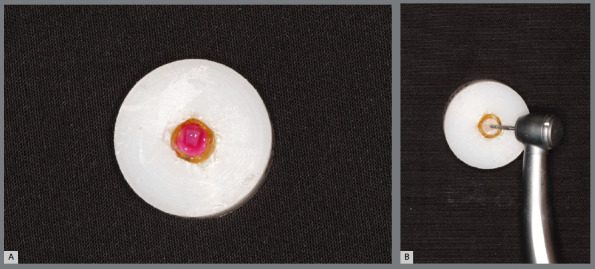
Whitening (A) and removal of the GIV attachment (B).

The specimens from both groups (Fig. 1) were subjected to two bleaching treatment sessions with an interval of 7 days, strictly following the manufacturer’s recommendations (Fig. 3). An in-office tooth whitening system with 35% hydrogen peroxide (Whiteness HP, FGM Dental products, Joinville, SC, Brazil) was used. During the 14 days of research, all specimens (n=80) were kept immersed in artificial saliva to ensure enamel rehydration.^14^ At the end of the analyses, the attachments were removed and a new color evaluation was carried out.

**Figure 4:**
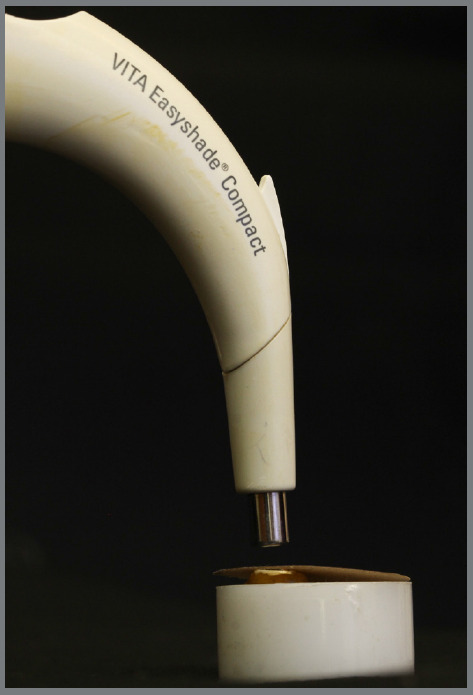
Portable digital spectrophotometer positioned perpendicularly to the attachment, through a prefabricated support, in the same light environment and an opaque black cardboard mask with a central opening window.

The specimens were subjected to color evaluation according to Table 1. The colorimetric reading was performed using a portable digital spectrophotometer VITA Easyshade^®^ Compact (Germany - Model DEASYC220), positioned perpendicular to the attachment, through a prefabricated support, in the same light environment and an opaque black cardboard mask with a central opening window was used (Fig. 4). The color was evaluated according to the LAB color scale of the Commission Internationale de l’Eclairage (CIE), relative to the D65 lighting standard, which divides the color by the mathematical process of the colorimetric curve into fields L*, a* and b*.^15^ Five measurements were carried out for each sample and the value obtained for each specimen (L*, a* and b*) corresponded to the average of these measurements. The color change (ΔE) was calculated by the equation ΔE=[(ΔL)² + (Δa)² + (Δb)²]1/2. To assess color change, ΔE* was converted to the National Bureau of Standards (NBS).^16^ ^-^ ^19^

Descriptive statistics (means and standard deviations) were used to present the ∆L, ∆a, ∆b, ∆E, and NBS for each study group. General linear models fit by ordinary least squares regression were used to test the effect of whitening, the use of attachment, and the interaction of both factors (whitening*attachment) on tooth color stability. Models’ assumptions were assessed by the Levene’s test for homogeneity of residual variances and by the test of Shapiro-Wilk for normality of residuals. All analyses were performed in JAMOVI (v. 1.2) using two-tailed tests and adopting a significance level of 5%.

## RESULTS

Descriptive statistics for parameters ∆L, ∆a, ∆b, ∆E, and NBS are presented in Table 2. The fitted models showed that both factors ‘tooth whitening’ and the ‘use of attachment’ have an effect on all parameters (while keeping constant the other variable) (Table 3). No interaction was observed between both factors in any of the implemented models. 

**Table 2:** Mean and standard deviation values of CIELAB parameters.

CIELAB parameters	Groups			
	GI	GII	GIII	GIV
∆L	-4.04 (5.82)	-1.34 (4.31)	0.84 (6.66)	3.98 (3.55)
∆a	-0.47 (1.68)	-2.74 (2.32)	0.80 (1.75)	-0.17 (2.23)
∆b	-3.81 (4.37)	-9.24 (4.79)	-0.50 (3.45)	-5.89 (4.00)
∆E	8.25 (4.09)	11.1 (4.17)	6.47 (4.11)	8.08 (4.26)
NBS	7.59 (3.77)	10.20 (3.84)	5.95 (3.78)	7.43 (3.92)

While the application of tooth whitening corresponds on average to an increase of 2.04 (95% CI: 0.33, 3.74; p=0.020) NBS units (greater color change), the use of attachment corresponds to a decrease of 2.20 (95% CI: -3.90, -0.50; p=0.012) NBS units (minor color change). Tooth whitening produced significantly higher values for ∆L (p=0.015) and lower values for ∆a and ∆b (p<0.001) compared to no whitening. On the other hand, the use of attachments generated significantly higher values for all parameters, ∆L, ∆a and ∆b (p<0.001) compared to not using attachment.

## DISCUSSION

The use of aligners has become popular among patients and orthodontists worldwide. In this context, a question arises: would the presence of these appliances interfere with tooth whitening? In the search for an answer to this question, the present study aimed to evaluate the effect of using 35% hydrogen peroxide on the color change of the enamel of bovine teeth with and without orthodontic appliances bonded to their surface.

The main findings of this study showed that the variables “whitening” and “attachments” were significant when crossed with the variables that analyze possible color changes (∆L, ∆a, ∆b, ∆E, and NBS). It shows that there was an effect on the samples of both the whitening application and the presence of attachments. Nevertheless, this scenario was not visualized when comparing a single model “tooth whitening and attachments”. Therefore, the whitening effect was independent of the presence of attachments. The results are in line with Silvestre et al^14^ in which they showed color changes in areas that were not directly exposed to the whitening agent, under the resin fragment, in order to confirm its diffusion potential.

As for the whitening effect, the variables ΔE and NBS showed a color change at the end of the study (ΔE=2.22 (0.36, 4.07); p=0.020* / NBS=2.04 (0.33, 3.74); p=0.020*). GII without attachment and with whitening showed a greater difference between the means in ΔE and NBS (ΔE=11.1 (4.17) / NBS=10.20 (3.84)) suggesting to be the group with the greatest visible color change (Table 2). The ∆L parameter showed an increase in the luminosity of the samples, suggesting teeth whitening (ΔL=2.92 (1.17, 0.59); p=0.015*) (Table 3). Dental whitening producing an increase of ∆L is already an expected finding. Regarding the variable Δa, the change in a* was toward green (Δa=-1.62 (-2.51, -0.72); p<0.001*) which confirms, once again, the whitening effect. The groups submitted to whitening (GII and GIV) also presented negative means, however, the group without attachment (GII) presented means farther from 0 (-2.74 (2.32)) compared to the group (GIV) that underwent whitening with bonded attachments (-0.17 (2.23)). Δb proved to be closer to the blue color (Δb= -5.41 (-7.28, -3.55); p<0.001*), and similarly to what happened with Δa, GII presented more negative means compared to GIV.

**Table 3:** Descriptives statistics of color stability values according to whitening, attachment and whitening*attachment.

Variables	CIELAB parameters									
	∆L			∆a		∆b		∆E		NBS	
	β (95% CI)	P value	β (95% CI)	P value	β (95% CI)	P value	β (95% CI)	P value	β (95% CI)	P value
Whitening	2.92 (1.17. 0.59)	0.015*	-1.62 (-2.51. -0.72)	<0.001*	-5.41 (-7.28. -3.55)	<0.001*	2.22 (0.36. 4.07)	0.020*	2.04 (0.33. 3.74)	0.020*
Attachment	5.10 (2.77. 7.43)	<0.001*	1.92 (1.02. 2.82)	<0.001*	3.33 (1.46. 5.19)	<0.001*	-2.39 (-4.24. -0.54)	0.012*	-2.20 (-3.90. -0.50)	0.012*
Whitening*Attachment	0.45 (-4.21. 5.11)	0.848	1.30 (-0.49. 3.10)	0.152	0.04 (-3.69. 3.76)	0.984	-1.21 (-4.92. 2.49)	0.517	-1.11 (-4.52. 2.30)	0.517

* statistically significant values

Regarding the effect of attachments, the variables ΔE and NBS showed color darkening at the end of the study (ΔE=-2.39 (-4.24, -0.54); p=0.012* / NBS=-2.20 (-3.90, -0.50); ​​p=0.012*). It is important to emphasize that the diamond bur used to remove the attachments may have influenced the increase in the variable. The group with attachment and whitening exhibited a greater difference between the means in ΔE and NBS when compared to GIII (with attachment and without whitening), suggesting a greater visible color change, as previously mentioned (Table 2). The fact that the use of attachments corresponds to a decrease in NBS can be explained by the adhesion of the composite resin to the tooth, decreasing the diffusion capacity of hydrogen peroxide. The ∆L parameter showed an increase in luminosity in all groups (ΔL=5.10 (2.77, 7.43); p<0.001*), but with greater representation in GIV (ΔL3.98 (3.55)). Regarding Δa, isolating the “attachment” variable, it presented positive values ​​suggesting proximity to red (Δa=1.92 (1.02, 2.82); p<0.001*), however, when analyzing the groups, the variable proved to be closer to the green in GIV (Δa=-0.17 (2.23)) and closer to red in GIII (Δa=0.80 (1.75)), which is completely predicted since GIII did not undergo whitening. Like Δa, Δb showed values ​​suggesting proximity to yellow (Δb=3.33 (1.46, 5.19); p<0.001*), in contrast when analyzing the groups with the presence of attachments, GIV (Δb=-5.89 (4.00)) exhibited a negative value farther from 0 in relation to GIII (Δb-0.50 (3.45)), which presupposes values ​​closer to blue.

When evaluating coordinates of L*, a* and b* separately, the ∆L parameter was represented with the highest mean by the group with attachment and with whitening, which, alone, suggests that it was the group with lighter teeth. Likewise, GIV showed a higher mean ∆L when compared to GIII (with attachment and without whitening) (Table 2). When evaluating only the L* coordinate, it is inferred that the attachment decreases the luminosity and consequently the whitening efficiency as predicted by Schlosser et al.^20^ When evaluating the variables Δa and Δb, the variable ∆a obtained the highest average in the group with attachment and without whitening, proving to be closer to the red spectrum. Group GII (without attachment and with whitening) had a lower average than group GI (without attachment and without whitening), suggesting that it was closer to green, which is in line with the fact that GII underwent whitening and GI did not. Following the same reasoning, GIV having a lower average than GIII. Similarly, ∆b also expressed the highest average in GIII. GII showed a lower average than GI, which suggests that GII is closer to blue, making it possible to infer that whitened bovine teeth are more translucent when compared to non-whitened ones. The same occurs when relating groups III and IV. And, although GIV showed clearing, GII (without attachment) showed the lowest average of the four groups.

With regard to the effectiveness of tooth whitening, a clinically visible color change is considered when the difference in the means of the ΔE values ​​is greater than 3.7 units^21^ The value of (L*) is the main attribute to be considered, as it represents the luminosity and clarity of the color.^22^ ^,^ ^23^ However, in the literature there is still no consensus on which of the parameters (L*, a* and b* or ΔE) is the best to evaluate the effectiveness of tooth whitening.^24^

The diffusion capacity of whiteners is something well supported in the literature and is due to the permeability present in dental tissues together with the low molecular weight and the ability of hydrogen peroxide to generate free radicals that act in several directions and degenerate pigments that cause darkening dental.^3^ ^,^ ^5^ ^,^ ^25^ ^-^ ^28^ Pinzan-Vercelino et al.^29^ evaluated the effectiveness of various whitening and bleaching products on orthodontic brackets bonded to bovine incisors and observed significant changes in tooth color when using a dental whitener containing 35% hydrogen peroxide.

Rego et al.^30^ assessed the bond strength of brackets bonded to premolars that had been bleached with 35% hydrogen peroxide. They found a significant decrease in bond strength when brackets were bonded 24 hours after whitening. However, after 7 days, no differences in bond strength were observed. Britto et al.^31^ examined the effect of desensitizing agents on the bond strength of ceramic brackets bonded to bovine enamel following the same whitening protocol and concluded that a whitening agent with calcium, when combined with desensitizing gel, enhances shear strength. Aristizábal, González, and McNamara^32^ evaluated a bonding protocol for metal brackets on bovine incisors after whitening with 35% hydrogen peroxide. They found that bonding immediately after whitening reduced shear bond strength, while treating the bleached enamel surface with 10% sodium ascorbate reversed the reduction in shear bond strength.

Although bovine teeth are routinely used in dental experiments because they have morphological characteristics similar to human tissue,^14^ an in vitro study does not faithfully simulate conditions in the oral cavity, which can cause variations in the results presented, pointing to a limitation of the study. Other factors capable of influencing are the thickness of the dentin related to the patient’s age^26^ and the initial color of the tooth, since young teeth generally have a lower degree of pigmentation.^33^ In in vitro studies, factors such as standardization, temperature and packaging were controlled,^14^ on the other hand factors such as salivation and oral hygiene cannot be monitored, which means another limitation for the study.

## CONCLUSION

It is concluded with this study that it is possible, in vitro, to promote enamel whitening with orthodontic attachments bonded on its surface with the use of 35% hydrogen peroxide, making possible the treatment with aligners concomitantly with dental whitening.
